# Comparison of large language models in generating patient handouts for the dermatology clinic: A blinded study

**DOI:** 10.1016/j.jdin.2024.02.010

**Published:** 2024-02-21

**Authors:** Crystal T. Chang, Iesha L. Ticknor, Jacob-Anthony Spinelli, Bhavnit K. Bhatia, Sangeeta Marwaha, Paradi Mirmirani, Anne M. Seidler, Jeremy R. Man, Patrick E. McCleskey

**Affiliations:** a Kaiser Permanente Bernard J. Tyson School of Medicine, Pasadena, California; b Touro University California, Vallejo, California; c Department of Dermatology, The Permanente Medical Group, Richmond, California; d Department of Dermatology, The Permanente Medical Group, Napa, California; e Department of Dermatology, The Permanente Medical Group, Vallejo, California; f Department of Dermatology, The Permanente Medical Group, Oakland, California; g Department of Dermatology, Southern California Permanente Medical Group, Los Angeles, California

**Keywords:** alopecia, artificial intelligence, ChatGPT, dermatitis, dyspigmentation, large language models, patient education

*To the Editor:* Large language models (LLMs) process probabilistic associations rather than meaning, raising reservations regarding clinical reliability and utility.^1^ From 2022 to 2023, multiple LLM chatbots, including ChatGPT (GPT 3.5; OpenAI), Bard (Google), and BingAI (GPT-4/Prometheus; OpenAI/Microsoft), were released. As patient handouts are associated with satisfaction and adherence, the ability to generate accurate, understandable handouts via LLMs would be invaluable for busy specialties such as dermatology.^2^

In this single-blinded study, ChatGPT, Bard, and BingAI were queried on 18 topics across 3 categories (dermatitis, alopecia, dyspigmentation) (total 54 handouts) using the prompt “Write a 1-page patient handout at a sixth grade reading level about [topic] including symptoms, diagnosis, prevention, and treatment.” Each handout received a Simple Measure of Gobbledygook (SMOG) readability score.^3^^,^^4^ SMOG is a validated, computer-generated score preferred for medical content; a SMOG score of 6 approximates sixth grade reading level (target for patient handouts according to the American Medical Association).^2^, ^3^, ^4^ Each handout was evaluated by 2 blinded nonmedical volunteers (pool of 5 raters) using the Patient Education Materials Assessment Tool (PEMAT),^5^ a validated measure of understandability; higher score indicates greater understandability. Lastly, 6 blinded attending dermatologists ranked 3 handouts from most to least preferred according to content and the least amount of time they would expect to spend revising them. Analysis was conducted in R. A generalized linear model was used to model PEMAT (Supplementary Data, available via Mendeley at https://data.mendeley.com/datasets/5ngxkzkdp9/3).

ChatGPT outperformed Bard and BingAI in the dermatologist ranking (% handouts ranked first 46.3% vs 15.7% and 38.0%, respectively; Table I, Supplementary Fig 1, available via Mendeley at https://data.mendeley.com/datasets/5ngxkzkdp9/3). ChatGPT was the most preferred for dermatitis and alopecia but slightly less preferred than BingAI on dyspigmentation.

**Table I:** ChatGPT, Google Bard, and BingAI were queried on 18 different topics from 3 categories (dermatitis, alopecia, and dyspigmentation) to generate a total of 54 unique handouts

	ChatGPT	Bard	BingAI	*P* value
SMOG grade level1∗				
Mean (SD)	8.80 (1.00)	7.39 (1.44)	6.72 (0.88)	ANOVA *P* = 4.47e-06∗∗∗ Pair-wise comparisons (Tukey-HSD): BingAI-Bard *P* = .182 ChatGPT-Bard *P* = .00141∗∗ ChatGPT-BingAI *P* = .0000034∗∗∗
Dermatitis†	8.47 (0.67)	8.23 (1.74)	6.58 (0.93)	
Alopecia‡	8.60 (1.38)	6.95 (0.99)	6.45 (0.94)	
Dyspigmentation§	9.33 (0.72)	7.00 (1.35)	7.12 (0.75)	
Mean nonmedical participant PEMAT understandability % (SD)	89.53% (10.65)	81.50% (9.87)	88.31% (9.16)	
Dermatitis†	88.00% (12.34)	78.67% (9.30)	88.83% (5.64)	GLM model: PEMAT ∼ Category + LLM + PEMAT rater‖ Simultaneous tests for general linear hypotheses (Tukey-HSD): BingAI-Bard *P* = .00749∗∗ Bard-ChatGPT *P* = .00131∗∗ BingAI-ChatGPT *P* = .870
Alopecia‡	86.50% (9.78)	83.00% (10.73)	87.08% (12.46)	
Dyspigmentation§	94.08% (8.84)	82.83% (9.75)	89.00% (8.80)	
*n* = 5 raters¶				
Board-certified dermatologist preference ranking (% ranked first)	46.3% (50/108)	15.7% (17/108)	38.0% (41/108)	NA
Dermatitis†	50.0% (18/36)	19.4% (7/36)	30.6% (11/36)	
Alopecia‡	47.2% (17/36)	13.9% (5/36)	38.9% (14/36)	
Dyspigmentation§	41.7% (15/36)	13.9% (5/36)	44.4% (16/36)	
*n* = 6 raters#				

*HSD,* Honestly significant difference; *LLM*, large language model; *PEMAT*, Patient Education Materials Assessment Tool; *SMOG*, Simple Measure of Gobbledygook.

∗ SMOG readability is calculated according to the formula grade level = sqrt (number of words ≥ 3 syllables) + 3.^3^

† Dermatitis category diagnoses included atopic dermatitis, seborrheic dermatitis, psoriasis, intertrigo, venous stasis dermatitis, and asteatotic dermatitis.

‡ Alopecia category diagnoses included telogen effluvium, alopecia areata, traction alopecia, central centrifugal cicatricial alopecia, lichen planopilaris, and androgenic alopecia.

§ Dyspigmentation category diagnoses included melasma, postinflammatory hyperpigmentation, vitiligo, progressive macular hypomelanosis, lichen planus pigmentosus, and exogenous ochronosis.

‖ With ChatGPT as the baseline, the coefficient for the ChatGPT-Bard term (within the LLM term) was the only statistically significant predictor of PEMAT score. For *P* values for other terms and additional data, please see the Supplementary Data, available via Mendeley at https://data.mendeley.com/datasets/5ngxkzkdp9/3).

¶ Each of the 54 handouts was scored using PEMAT by 2 independent nonmedical raters. Four raters scored 21 handouts (7 from each of 3 categories). One rater scored 24 handouts (8 from each of 3 categories).

# Each of the 54 handouts was compared to the 2 other handouts generated by other LLMs on the same topic and ranked by preference (first, second, third) by 6 board-certified dermatologists. Each dermatologist reviewed and ranked all 54 handouts.

ChatGPT and BingAI both outperformed Bard on the PEMAT score for understandability (ChatGPT-Bard *P* < .001, Tukey-honestly significant difference for multiple comparisons; Table I, Supplementary Fig 2, available via Mendeley at https://data.mendeley.com/datasets/5ngxkzkdp9/3). ChatGPT was not significantly different from BingAI (*P* = .62). LLM was the only statistically significant predictor of PEMAT score (category of handout and PEMAT rater were not significant).

Mean SMOG grade level was lowest for BingAI, followed by Bard and ChatGPT (6.72 vs 7.39 and 8.80; Tukey-honestly significant difference *P* < .01 for ChatGPT-Bard and ChatGPT-BingAI; Table I, Supplementary Fig 3, available via Mendeley at https://data.mendeley.com/datasets/5ngxkzkdp9/3).

ChatGPT produced the handouts that were most preferred by the dermatologists. ChatGPT and BingAI scored higher than Bard on understandability based on PEMAT. BingAI most closely approximated sixth grade reading level using SMOG; however, the variability between sixth and eighth grade reading level by SMOG, which has been criticized for its penalty of multisyllabic words, may not be clinically significant.^4^

Limitations of this study include the small number of reviewers and evaluation within a single health system for the dermatologist score (readability and understandability were not evaluated within a single health system). Additionally, only the first output for each prompt was evaluated with the full set of metrics; however, we separately qualitatively assessed a subset of prompts run multiple times and did not find much stochastic variability.

Though LLM-generated handouts may need revision for accuracy and practice personalization, the generally high scores across multiple metrics indicate that, with oversight, LLMs can produce understandable, accurate handouts. As models continue evolving rapidly, clinicians will increasingly need to assess the utility of new software updates and whether educational tools influence patient-centered outcomes.

## Conflicts of interest

None disclosed.
